# Ultrasound-guided microwave ablation for small thyroid nodules with RAS mutation: a pilot study

**DOI:** 10.3389/fonc.2026.1773527

**Published:** 2026-04-22

**Authors:** Fengyun Cheng, Yujiang Li, Ruixue Huang, Zhiwei He, Yueting Zhao, Xiaoqiu Chu, Chao Liu, Shuhang Xu

**Affiliations:** 1 Endocrine and Diabetes Center, Affiliated Hospital of Integrated Traditional Chinese and Western Medicine, Nanjing University of Chinese Medicine; Jiangsu Province Academy of Traditional Chinese Medicine, Nanjing, China; 2 Department of Endocrinology, Sheyang County People' s Hospital, Yancheng, China; 3 Key Laboratory of TCM Syndrome and Treatment of Yingbing (Thyroid Disease) of State Administration of Traditional Chinese Medicine, Jiangsu Province Academy of Traditional Chinese Medicine, Nanjing, China

**Keywords:** microwave ablation, nodule volume, RAS mutation, thyroid nodule, volume reduction rate

## Abstract

**Objective:**

The clinical management of RAS-mutated nodules faces significant challenges. This study aimed to explore the efficacy of microwave ablation (MWA) for the treatment of thyroid nodules ≤ 2 cm with isolated RAS gene mutations.

**Methods:**

This retrospective study was conducted between February 2022 and July 2024. A total of 23 patients with small RAS-mutated thyroid nodules who underwent MWA and completed at least 12 months of follow-up were enrolled. Data on nodule volume, volume reduction rate (VRR), and MWA-related adverse events were collected. Intra-group comparisons were performed using the Friedman rank sum test with *post-hoc* pairwise comparisons. Factors affecting VRR were analyzed using linear regression. ROC analysis was conducted as an exploratory approach to determined the optimal diameter cut-off for predicting complete regression.

**Results:**

The cohort included 4 males (17.39%) and 19 females (82.61%), with a mean age of 45.35 ± 14.65 years. The median preoperative maximum diameter and volume were 6.20 (5.30-8.70) mm and 81.16 (46.12-256.56) mm³, respectively. All 23 patients completed ≥ 12 months of follow-up, with 6 patients reaching the 24-month follow-up. After an initial transient enlargement, the nodules showed sustained shrinkage. At the 12-month follow-up, the median VRR was 11.84% (P < 0.001), and the complete regression rate was 13.0%. By 24 months, the median VRR had reached 100%, with 83.33% complete regression (5/6). Nodules < 6 mm had higher regression rates. No local recurrence or distant metastasis occurred during the available follow-up period.

**Conclusion:**

Preliminary findings suggest that MWA may have favorable short-term efficacy and safety in the treatment of small RAS-mutated thyroid nodules.

## Introduction

RAS mutations, including HRAS, KRAS, and NRAS subtypes are common molecular genetic variations in thyroid tumors (1, 2), promoting abnormal cell proliferation and differentiation, and contributing to tumorigenesis (3). RAS mutations occur in both malignant and benign thyroid lesions. They are most common in follicular variant of papillary thyroid carcinoma (FVPTC) and follicular thyroid carcinoma (FTC), accounting for approximately 30%-45%, but also present in benign thyroid diseases, including follicular thyroid adenoma (FTA) and goiter (4–7). The shared follicular cell origin and RAS-driven hyperplasia result in low sensitivity of RAS mutations for preoperative differentiation between benign and malignant lesions (8–11). Nabhan et al. (12) found widely varying positive predictive values of RAS across different Bethesda categories. Consequently, the management of RAS-mutated thyroid nodules poses a considerable clinical dilemma.

Currently, surgery remains the primary treatment for these lesions (13), combined with neck lymph node dissection if necessary. However, surgical intervention carries the risk of overtreatment, particularly for preoperatively indeterminate RAS-mutated nodules, potentially causing unnecessary organ damage and functional impairments. Some studies suggest that RAS-mutated nodules under active surveillance often show indolent behavior with low progression risk. However, the possibility of malignancy still exists (14, 15) and can increase patient anxiety levels. Consequently, balancing the risks of “overtreatment” against the uncertainties of “active surveillance” has become a major clinical challenge. Thus, there is a growing need to explore minimally invasive, organ-preserving treatments that ensure both safety and efficacy.

Recent advances have established microwave ablation (MWA) as a viable option for thyroid nodules and low-risk papillary thyroid carcinoma (PTC) (16, 17). For papillary thyroid microcarcinoma (PTMC) with a diameter of ≤ 1 cm and without metastasis, the efficacy of MWA is comparable to that of surgery (18, 19). The latest expert consensus (20) supports expanding ablation to T1bN0M0 PTC with a diameter of > 1 cm and ≤ 2 cm, and studies report comparable outcomes in T1a and T1b groups after MWA without differences in disease progression or metastasis (21), supporting MWA as an effective treatment for selected PTC up to 2 cm.

There is a lack of relevant evidence regarding the efficacy and safety of MWA for RAS-mutated thyroid nodules with a diameter of ≤ 2 cm. Therefore, this study aimed to evaluate the clinical value of MWA in the management of such nodules and to provide an evidence-based basis for optimizing treatment strategies.

## Methods

### Data source and study population

This study was conducted in accordance with the ethical principles of the Declaration of Helsinki 2013 and received ethical approval and waiver of consent from Affiliated Hospital of Integrated Traditional Chinese and Western Medicine, Nanjing University of Chinese Medicine (Ethics Approval No. 2024-LWKYZ-084). All patients signed an informed consent form and clearly understood the treatment plan. This was a retrospective, single-centre, cohort study. Patients with RAS-mutated thyroid nodules ≤ 2 cm who underwent microwave ablation (MWA) at the Affiliated Hospital of Integrated Traditional Chinese and Western Medicine from February 2022 to July 2024 were enrolled.

Inclusion Criteria were as follows: (i) aged 18 - 70 years; (ii) maximum diameter of the nodule ≤ 2.00 cm; (iii)Presence of NRAS, KRAS, or HRAS mutations confirmed by gene testing, without other gene mutations; (iv) imaging examinations showing no evidence of capsular invasion, extrathyroidal extension, recurrent laryngeal nerve/tracheal invasion, cervical lymph node metastasis, or distant metastasis; (v) completion of ≥ 12 months of follow-up after MWA treatment, with complete follow-up data; (vi) no history of neck radiotherapy or thyroid surgery; (vii) the patient’s willingness to undergo fine-needle aspiration cytology (FNAC) combined with molecular diagnosis for clarifying the nature of the nodule; and (viii) patients selected MWA with full informed consent, declining both surgical intervention and long-term surveillance.

Exclusion Criteria were as follows: (i) comorbidity with other malignant tumors; (ii) allergy to the drugs used in this study; (iii) presence of severe bleeding tendency (prothrombin time > 18 s, platelet count < 50×10^9^/L) or coagulation dysfunction; (iv) comorbidity with systemic infection, high fever (body temperature > 38.5°C), or significant abnormal white blood cell count (< 3×10^9^/L or > 15×10^9^/L); (v) presence of severe dysfunction of major organs such as heart, liver, and kidney; (vi) pregnancy or lactation; (vii) incomplete clinical data or loss to follow-up during the follow-up period; and (viii) other conditions deemed unsuitable for inclusion in this study by the researchers.

### Molecular testing

Molecular testing was performed as described previously (22). Genomic DNA was extracted from FNA samples using a commercial kit (Qiaamp DNA Mini Kit, Qiagen). Sequencing libraries were prepared via a one-step multiplex PCR approach, amplifying a custom panel targeting variants in 14 key thyroid cancer driver genes (e.g., BRAF, NRAS, TERT) and 21 types of gene rearrangements. The amplicons were purified, quantified, and sequenced on an Ion Proton system (Thermo Fisher Scientific). Reads were aligned to the hg19 reference genome using Bowtie2, and variant calling was performed using Samtools and custom Perl scripts.

### Outcome measures

#### Preoperative baseline assessment

A Siemens ACUSON S2000 color Doppler ultrasound system was used for standardized thyroid nodule evaluation. An expert with over 10 years of experience in thyroid ultrasound performed image acquisition and analysis, recording nodule characteristics including: maximum diameter (three-dimensional orthogonal dimensions a, b, c), internal structure (solid/mixed/cystic), echogenicity (hypo-/iso-/hyperechoic), margin clarity (clear/unclear), aspect ratio (≥1 or <1), and calcification type (microcalcification/macrocalcification/none). Baseline patient data including sex, age, past medical history, and thyroid function indicators were collected. Preoperative chest and enhanced neck CT examinations were performed to confirm the absence of lung metastasis and cervical lymph node metastasis in patients. All patients underwent preoperative electronic laryngoscopy to evaluate vocal cord movement function and structural integrity and to rule out recurrent laryngeal nerve injury.

#### Intraoperative monitoring indicators

Ablation power, ablation time, and ablation needle puncture path were recorded during the operation. The energy absorption per unit volume of the tumor (E) was calculated using the following formula: E = ablation time (s) × power (W)/ablation volume (ml) [Unit: J/ml]. Immediately after ablation, contrast-enhanced ultrasound was performed to observe blood perfusion in the ablated area and evaluate the ablation efficacy.

#### Postoperative follow-up indicators

Patients were followed up at 1 day, 1, 3, 6, 12, and 24 months postoperatively. The following items were assessed: (i) Thyroid function: serum levels of free triiodothyronine (FT3), free thyroxine (FT4), thyroid-stimulating hormone (TSH), thyroglobulin (Tg), and thyroglobulin antibody (TgAb). (ii) Ablation lesion volume: measured using three-dimensional diameters (a, b, c) of the nodule, and calculated as V = abcπ/6 [9]. (iii) Initial ablation ratio (IAR): evaluated with contrast-enhanced ultrasound. IAR (%) = volume of the non-enhanced area immediately after ablation/preoperative nodule volume × 100%. (iv) VRR: calculated using the formula: VRR (%) = (preoperative volume - postoperative volume)/preoperative volume × 100%. (v) Complete disappearance of the nodule: defined as no detectable nodule or ablation lesion on ultrasound. The complete regression rate was defined as the proportion of nodules with complete disappearance. (vi) Safety: complications were recorded and graded according to the Classification System of the Cardiovascular and Interventional Radiological Society of Europe (CIRSE) (23).

### MWA

An MWA system (KY-2000; Canyon Medical) was used to perform ultrasound-guided MWA. The patient was positioned supine with the neck exposed. Following disinfection and sterile draping, local anesthesia with 1% lidocaine was administered under ultrasound guidance. Hydrodissection was performed using normal saline to isolate the thyroid capsule from surrounding critical structures.

The ablation procedure was conducted as previously described (24). Under continuous ultrasound guidance, the microwave ablation needle was advanced along a safe path to avoid vital cervical anatomy. Ablation was conducted until the entire nodule and a 3-mm margin were encompassed by a hyperechoic area. The needle tract was coagulated during withdrawal using residual heat to minimize seeding risk. Post-ablation color Doppler ultrasound confirmed the absence of blood flow within the treated area before concluding the procedure.

### Statistical analysis

SPSS 27.0 and GraphPad Prism 10 were used for data analysis and visual presentation. Continuous data were assessed for normality using the Shapiro-Wilk test. Normally distributed data are presented as mean ± standard deviation (x ± s), non-normal data as median (interquartile range) [M (P25, P75)], and categorical data as number (percentage) [n (%)]. Intragroup comparisons were conducted using the Friedman test with *post-hoc* pairwise analyses. Receiver operating characteristic (ROC) curve analysis was used as an exploratory approach to determine cutoff values. The complete remission rate was analyzed with Kaplan-Meier curves and compared using the log-rank test. Factors influencing volume reduction rate (VRR) were screened by univariate linear regression (exploratory analysis). A p-value < 0.05 was considered statistically significant.

## Results

### Baseline characteristics

A total of 23 patients (with 23 RAS-mutated thyroid nodules) were finally included. Among them, there were 4 male patients (17.39%) with a mean age of 46.47 years and 19 female patients (82.61%) with a mean age of 40 years; the overall mean age of all patients was 45.35 ± 14.65 years. All patients underwent ≥ 12-month follow-up. The median maximum diameter of the nodules was 6.20 (5.30, 8.70) mm, and the median volume was 81.16(46.12, 256.56) mm3. Among the 23 nodules, 12 (52.17%) had NRAS mutations, 7 (30.43%) had KRAS mutations, and 4 (17.39%) had HRAS mutations. The median preoperative TSH, FT3, FT4 level was 2.06 (1.45, 2.63) mIU/L, 4.77 (4.48, 5.00)pmol/L, 17.29 (16.52, 19.60) pmol/L, all of which were within the normal reference range.

Ultrasonography revealed that all 23 nodules were solid and had diameters of ≤ 2 cm. Among them, 1 (4.35%) was classified as C-TIRADS category 3, 14 (60.87%) as C-TIRADS category 4a, 6 (26.09%) as C-TIRADS category 4b, and 2 (8.70%) as C-TIRADS category 4c. All 23 nodules underwent fine-needle aspiration cytology (FNAC): 14 (60.87%) were categorized as Bethesda class I, 3 (13.04%) as Bethesda class III, 4 (17.39%) as Bethesda class IV, and 2 (8.70%) as Bethesda class V (**Table 1**).

**Table 1:** Baseline characteristics of 23 patients with 23 nodules with RAS-mutation.

Characteristic	Value
Patient/Number of nodules	23
Sex	19/4
Female	19(82.61%)
Male	4(17.39%)
Age (yr)	45.35 ± 14.65
Maximum diameter (mm)	6.2(5.3, 8.7)
Baseline volume (mm^3^)	81.16(46.12, 256.56)
Thyroid nodule mutation	
KRAS	7(30.43%)
NRAS	12(52.17%)
HRAS	4(17.39%)
Bethesda category at diagnosis	
Bethesda I	14(60.87%)
Bethesda III	3(13.04%)
Bethesda IV	4(17.39%)
Bethesda V	2(8.70%)
Ultrasonographic features at diagnosis	
Aspect ratio	
>1	3(13.04%)
≤1	20(86.96%)
Nodule structure	
Solid	23(100%)
Echogenicity	
Hypoechoic	13(56.52%)
Isoechoic	10(43.48%)
Borders	
Regular	14(60.87%)
Irrregular	9(39.13%)
Calcification	
Microcalcifications	6(26.09%)
None calcification	17(73.91%)
C-TIRADS categories	
3	1(4.35%)
4a	14(60.87%)
4b	6(26.09%)
4c	2(8.70%)
Thyroid function	
TSH (mIU/L)	2.06(1.45, 2.63)
FT4 (pmol/L)	17.29(16.52, 19.60)
FT3 (pmol/L)	4.77(4.48, 5.00)

C-TIRADS, China Thyroid Imaging Reporting and Data System; TSH, Thyroid-Stimulating Hormone; FT3, Free Triiodothyronine; FT4, Free Thyroxine.

### Changes in volume and VRR of RAS-mutated nodules

**Figure 1:**
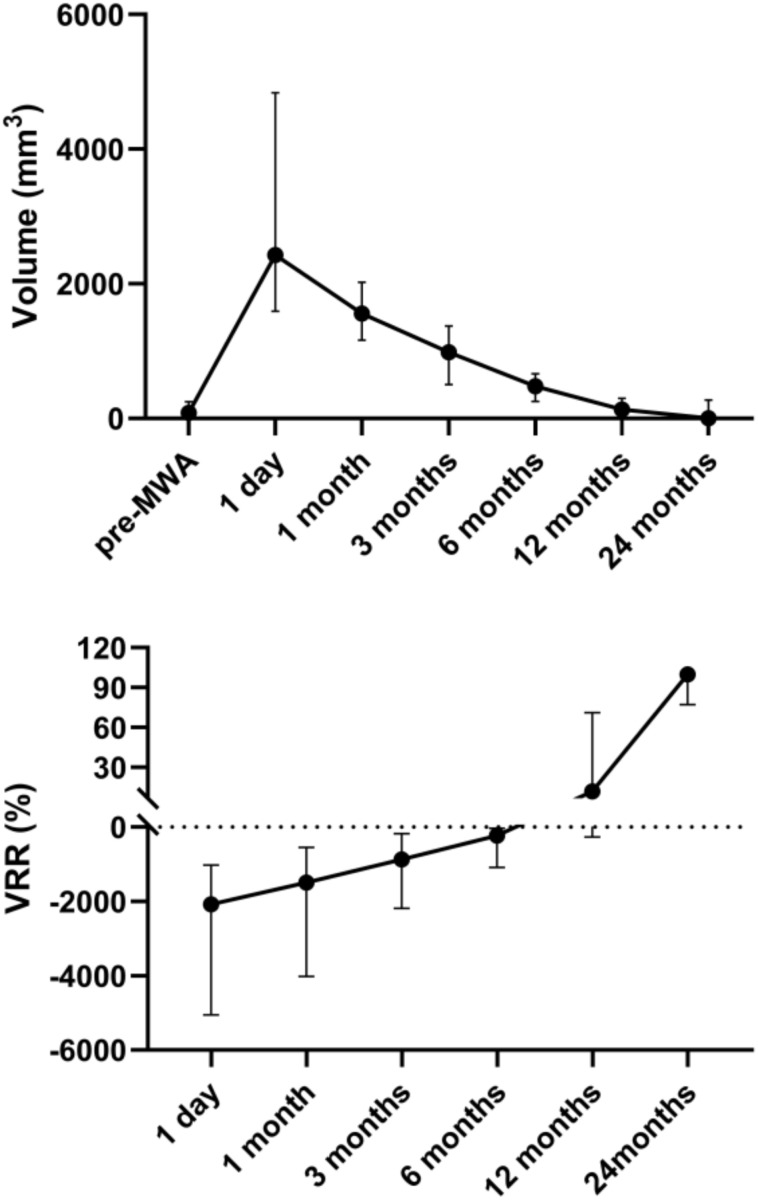
Changes in nodule volume and VRR before and after MWA. VRR, volume reduction rate; MWA, microwave ablation.

Nodule volume after MWA exhibited a transient increase followed by progressive decrease. The median volume on postoperative day 1 was significantly elevated compared to baseline (*P* = 0.044), then progressively declined at 1, 3, 6, 12, and 24 months. Volume returned to a level not significantly different from baseline by 3 months (*P* = 1.000). The median VRR showed a concordant trend, remaining negative at early timepoints and turning positive by 12 months with 11.84%, reaching 100% at 24 months (based on 6 patients who completed 24-month follow-up). *Post-hoc* pairwise comparisons indicated that the VRR at 24 months postoperatively was significantly higher compared to that on postoperative day 1 (*P* = 0.001) (**Table 2**; **Figure 1**).

**Table 2:** Changes in nodule volume and VRR during follow-up after MWA.

Follow-up	Volume (mm^3^)	Adjusted P	VRR (%)	Adjusted P
1 day	2428.33 (1505.35, 4997.98)	0.044	-2078.48 (-5061.29, -1024.76)	–
1month	1555.09 (853.00, 2699.41)	0.339	-1496.87 (-4017.32, -546.53)	1.000
3month	980.00 (418.88, 1552.11)	1.000	-870.11 (-2190.89, -176.13)	1.000
6month	475.01 (186.61, 854.52)	1.000	-236.73 (-1092.04, -38.12)	0.203
12month	130.90 (22.20, 329.87)	1.000	11.84 (-266.92, 71.08)	0.014
24month	0.000(0.000, 68.0675)	1.000	100(100, 100)	0.001

VRR, volume reduction rate.

**Figure 2:**
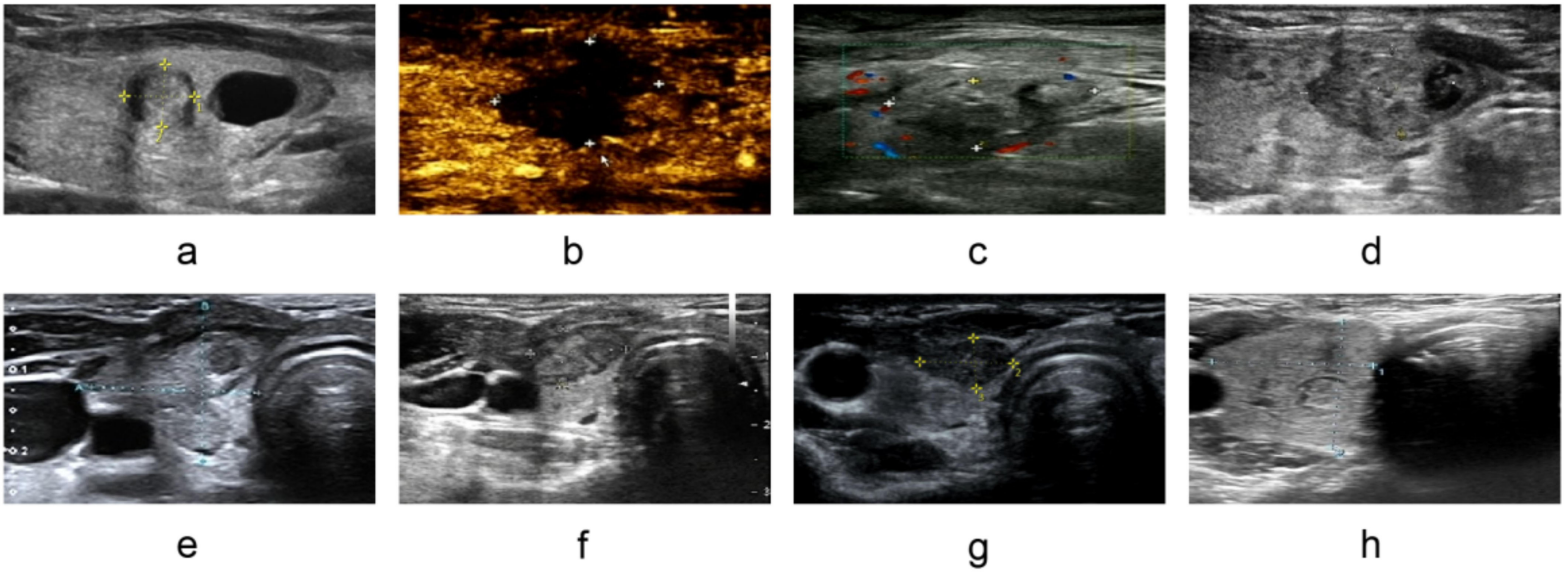
Ultrasonograms of a 24-year-old female patient with 24 months of follow-up. **(a)** Preoperative, **(b)** Contrast-enhanced ultrasound immediately after ablation, **(c)** 1 day postoperatively, **(d)** 1 month postoperatively, **(e)** 3 months postoperatively, **(f)** 6 months postoperatively, **(g)** 12 months Postoperatively, **(h)** 24 months Postoperatively.

The nodule disappearance rate gradually increased with the prolongation of follow-up period. At 1 month after surgery, 1 nodule (4.35%) disappeared on imaging; at 12 months after surgery, 3 nodules (13.04%) disappeared; and by 24 months after surgery, 5 out of 6 nodules (83.33%) disappeared (**Supplementary Table 1**). Typical ultrasonographic images show that the nodule gradually shrank after MWA and eventually disappeared at 24 months, demonstrating a significant ablation effect (**Figure 2**).

### Analysis of factors influencing the efficacy of MWA

Clinical data were included in a linear regression model as an exploratory analysis to explore factors affecting VRR. The results suggested that age, postoperative contrast volume, and IAR were negatively correlated with VRR, whereas female sex, nodule volume, power per unit volume, and preoperative maximum diameter were positively correlated with VRR (**Table 3**). However, none of these correlations reached statistical significance (*P*>0.05).

**Table 3:** Liner regression analysis of factors affecting VRR.

Factors	linear regression analysis		
	B	95% CI	P
Age (years)	-3.082	-35.909, 29.746	0.847
Gender	-268.393	-1504.125, 967.338	0.656
Maximum diameter (mm)	850.418	-448.059, 2148.895	0.188
Volume (mm^3^)	992.819	-1208.578, 3194.215	0.359
Postoperative contrast volume(mm^3^)	-47.310	-116.810, 22.199	0.171
Energy per unit volume(J/ml)	0.014	-0.042, 0.070	0.604
Gene	236.905	-374.575, 848.386	0.429
IAR(%)	-2.403	-5.572, 0.765	0.129

IAR, Initial ablation ratio; VRR, volume reduction rate.

**Figure 3:**
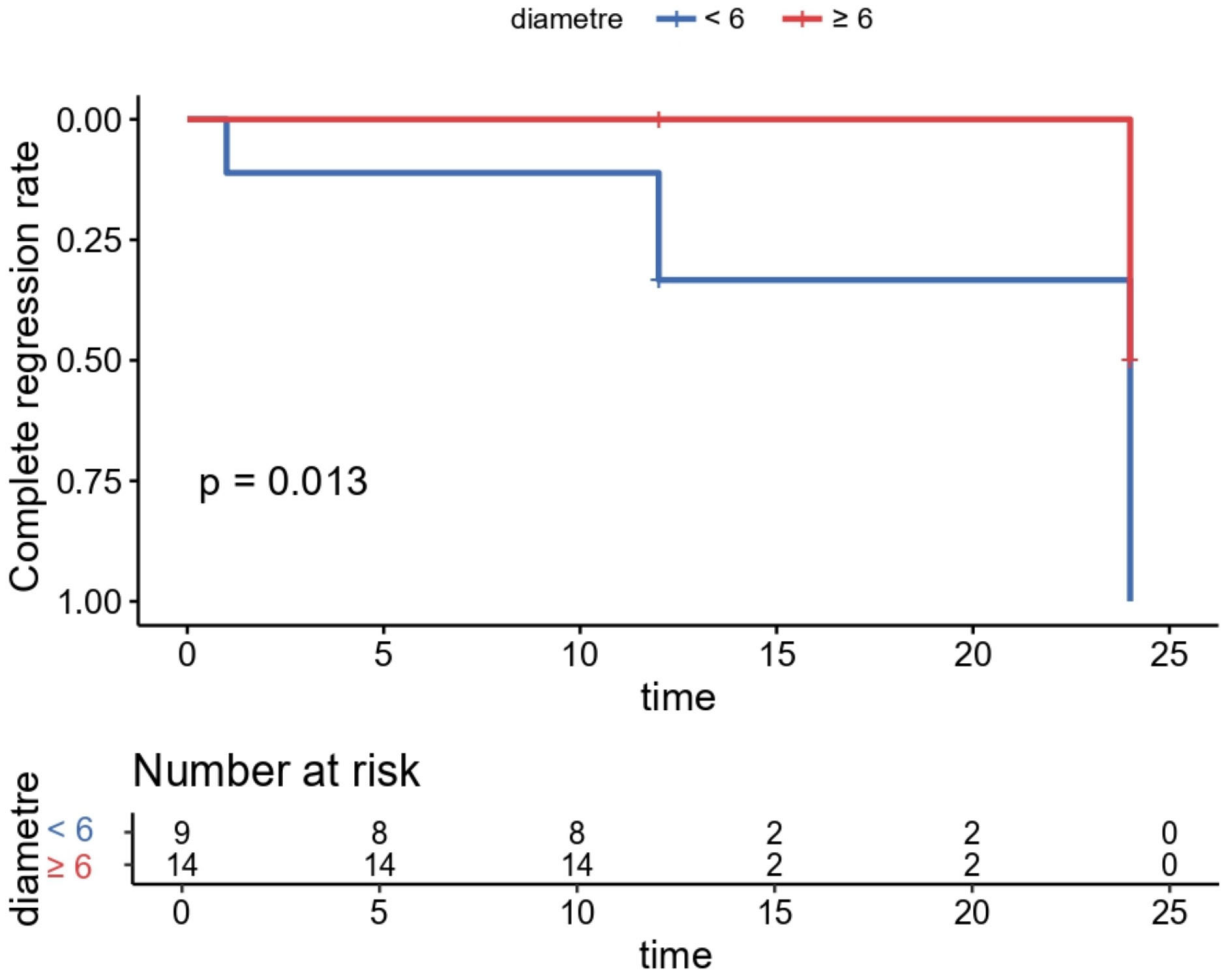
The rate of complete disappearance of nodules at each follow-up visit. MWA, microwave ablation.

ROC curve analysis, conducted as an exploratory approach, using “nodule disappearance” as the outcome indicator yielded a cutoff value of 6 mm for preoperative maximum diameter, with an AUC of 0.745, suggesting a potential higher likelihood of disappearance in nodules < 6 mm. However, the result was not statistically significant (*P* = 0.08). When patients were grouped by this 6-mm cutoff, Kaplan-Meier analysis with log-rank test showed a significantly higher complete regression rate in the smaller-nodule group (*P* = 0.013) (**Figure 3**). These exploratory findings should be interpreted cautiously due to the small sample size of the study.

### Safety

No tracheal, esophageal, or vascular injuries occurred in any of the patients after MWA. Additionally, there were no cases of tissue swelling, skin burns at the ablation site, or thyroid dysfunction during the available follow-up period. Only 1 patient developed hoarseness during the follow-up period, which was rated as grade III according to the CIRSE classification. The symptom was relieved after 1 month of symptomatic treatment. Imaging examinations showed no signs of metastasis or recurrence in any of the nodules during the study postoperative follow-up period.

## Discussion

Our retrospective study suggested that small RAS-mutated nodules treated with MWA showed progressive absorption over time. At the 24-month follow-up, a complete regression rate of 83.33% (5/6) was achieved (based on 6 patients who completed 24-month follow-up), with no recurrence, lymph node involvement, or distant metastasis observed during the entire follow-up period of this study. These findings suggest that MWA may be a potentially promising treatment option for these nodules, though this conclusion should be interpreted cautiously given the small sample size and limited follow-up completeness.

No previous studies have specifically investigated MWA for the treatment of small RAS-mutated thyroid nodules. However, the clinical characteristics of the included nodules were somewhat similar to those of low-risk papillary PTC. Consistent with these studies (25, 26), volumes at 1, 3, 6, 12, and 24 months were significantly lower than on postoperative day 1. This pattern is due to extended ablation. In low-risk thyroid tumors, optimizing the ablation range helps reduce local recurrence and improve treatment efficacy (27). Therefore, an extended ablation protocol was also applied to these small RAS-mutated nodules in this study. VRR, an objective indicator for quantifying nodule absorption efficiency, mirrored volume changes. This result suggests that RAS-mutated thyroid nodules may gradually disappear with extended follow-up, but further validation with larger sample sizes and longer follow-up is needed.

During the follow-up period, the complete tumor disappearance rate in this study was 26.09% (6/23), which was lower than the 84.5–99.37% reported in previous studies on PTMC after ablation (28, 29). It should be noted that only 6 patients in this study completed the 24-month follow-up, among whom 5 had complete disappearance of the ablation zone. The above results suggest that tumors tend to gradually disappear with prolonged follow-up. Whether RAS mutation itself may be a potential factor influencing nodule disappearance is still unknown. Notably, this study found that nodules with a preoperative maximum diameter of ≤ 6 mm showed a higher rate of complete disappearance. Among the 6 patients with complete nodule disappearance, only 1 had a preoperative maximum nodule diameter of > 6 mm, and the remaining 5 had a diameter of < 6 mm. This is consistent with the conclusion of our previous study that the maximum tumor diameter affects the disappearance rate of PTMC after ablation (30), and may indicate that smaller lesions are associated with a higher likelihood of complete regression following ablation.

This study investigated factors influencing ablation zone absorption after MWA in patients with RAS-mutated thyroid nodules. Sim et al. (31) reported that IAR strongly correlates with VRR in PTMC. Similarly, in this study, IAR showed a trend toward a negative correlation with VRR in RAS-mutated nodules, possibly due to protein denaturation making the ablation zone less absorbable (32). Additionally, age exhibited a trend toward a negative correlation with VRR, consistent with earlier studies (33), which may reflect reduced tissue repair and absorption capacity in older patients (34). However, none of these correlations reached statistical significance (*P* > 0.05), likely due to the limited sample size and insufficient statistical power.

No local recurrence or lymph node metastasis was observed during follow-up, aligning with previous ablation studies on low-risk thyroid tumors (16, 35), supporting the short-term safety and efficacy of MWA for these nodules. This may be attributed to the extended ablation strategy and the indolent nature of RAS-mutated nodules. It should be noted, however, that the median follow-up was only 12 months, which is inadequate to evaluate long-term outcomes.

The main strength of this study is that it provides preliminary evidence for the short-term efficacy of thermal ablation in treating RAS-mutated thyroid nodules ≤ 2 cm in maximum diameter, which may offer a potential alternative treatment option for such lesions. However, this study still has certain limitations. First, it was a single-center retrospective study with a small sample size, which may have introduced a selection bias. Second, the follow-up time was short, making it impossible to evaluate the risk of long-term recurrence or distant metastasis. Third, postoperative chest CT was not performed to monitor for metastatic lesions. Fourth, there was a lack of direct comparison with both the surgical group and the active surveillance group. Finally, the enrolled patients were mainly classified as Bethesda I class, with relatively few Bethesda III, IV, and V classes, which limits the generalization of the results to higher-risk nodules. Therefore, large-scale multicenter prospective studies with longer follow-up and more enrollment of Bethesda III, IV, and V nodules are needed to validate the long-term safety and efficacy of MWA for RAS-mutated thyroid nodules.

## Conclusions

MWA appears to achieve favorable short-term efficacy and safety in the treatment of thyroid nodules ≤ 2 cm with isolated RAS gene mutations, and may represent a reasonable alternative to surgery. Larger-scale studies with longer follow-up are warranted to verify its long-term outcomes.

## Data Availability

Publicly available datasets were analyzed in this study. This data can be found here: https://www.jianguoyun.com/p/DehwBekQ9tCXDhjV86EGIAA.
