# Supplementary material for: Ultrasound-guided microwave ablation for small thyroid nodules with RAS mutation: a pilot study
Source: Front Oncol. 2026 Apr 22;16:1773527. doi: 10.3389/fonc.2026.1773527 (PMC13147193; doi:10.3389/fonc.2026.1773527)
Supplement: Supplementary file 1 [file Table1.docx]

Supplementary Table 1. Follow-up data on patients

| Patient NO. | TIRADS | TBSRTC | Volume (mm^3^) | | | | | | |
| --- | --- | --- | --- | --- | --- | --- | --- | --- | --- |
|  |  |  | Pre-MWA | 1st Day | 1st Month | 3rd Month | 6th Month | 12th Month | 24th Month |
| 1 | 4a | Ⅳ | 65.93 | 1591.74 | 1319.47 | 835.67 | 431.97 | 241.90 | Disappear |
| 2 | 4b | Ⅰ | 57.78 | 2513.03 | Disappear | Disappear | Disappear | Disappear | Disappear |
| 3 | 4a | Ⅲ | 40.07 | 1193.81 | 1539.38 | 1075.00 | 611.00 | 147.24 | Disappear |
| 4 | 4c | Ⅰ | 53.95 | 804.25 | 604.76 | 418.88 | 175.93 | 85.33 | - |
| 5 | 4a | Ⅴ | 112.28 | 2343.62 | 1935.23 | 1522.11 | 854.52 | 329.87 | Disappear |
| 6 | 4a | Ⅳ | 107.54 | 1843.07 | 2022.40 | 1130.98 | 186.61 | Disappear | Disappear |
| 7 | 4a | Ⅰ | 299.14 | 3364.65 | 1979.21 | 1370.78 | 664.68 | 469.00 | 272.27 |
| 8 | 4b | Ⅲ | 101.48 | 12775.52 | 4691.46 | 3049.45 | 1926.60 | 807.85 | - |
| 9 | 4b | Ⅰ | 81.16 | 4188.80 | 7791.17 | 7225.68 | 475.01 | 29.38 | - |
| 10 | 4b | Ⅰ | 53.18 | 1246.17 | 687.63 | 515.91 | 251.33 | 31.42 | - |
| 11 | 4a | Ⅳ | 19.84 | 9206.19 | 3939.57 | 1869.25 | 1407.44 | 898.50 | - |
| 12 | 4b | Ⅰ | 249.97 | 2001.20 | 1566.61 | 366.52 | 402.37 | 22.20 | - |
| 13 | 4c | Ⅰ | 94.60 | 1963.50 | 1163.96 | 703.72 | 501.00 | 296.59 | - |
| 14 | 4a | Ⅰ | 51.41 | 1602.22 | 821.00 | 376.99 | 117.29 | 15.22 | - |
| 15 | 4a | Ⅰ | 956.08 | 2670.36 | 1319.47 | 1099.56 | 335.10 | 276.46 | - |
| 16 | 3 | Ⅲ | 46.12 | 1004.73 | 853.00 | 702.00 | 549.78 | 115.42 | - |
| 17 | 4a | Ⅰ | 469.15 | 2945.25 | 1470.27 | 980.00 | 490.09 | 130.90 | - |
| 18 | 4a | Ⅰ | 372.71 | 947.80 | 1555.09 | 502.66 | 315.00 | 125.66 | - |
| 19 | 4a | Ⅳ | 15.93 | - | 157.08 | 43.98 | 22.00 | Disappear | - |
| 20 | 4a | Ⅰ | 28.20 | 6531.39 | 5073.68 | 3675.67 | 2580.30 | 725.71 | - |
| 21 | 4b | Ⅴ | 11.07 | 5477.74 | 2699.41 | 244.07 | 7.72 | 5.60 | - |
| 22 | 4a | Ⅰ | 256.56 | 4838.06 | 1658.76 | 1068.14 | 863.94 | 226.20 | - |
| 23 | 4a | Ⅰ | 297.23 | 18397.21 | 12238.10 | 6809.33 | 4822.84 | 4538.11 | - |

C-TIRADS, China Thyroid Imaging Reporting and Data System; TBSRTC, The Bethesda System for Reporting Thyroid Cytopathology.
